# Comparative Pacing Profile and Chronometric Performance in Elite Swimmers with Intellectual Impairments and Able-Bodied Athletes

**DOI:** 10.3390/life14121623

**Published:** 2024-12-07

**Authors:** Luca Puce, Piotr Zmijewsk, Nicola Luigi Bragazzi, Carlo Trompetto

**Affiliations:** 1 Department of Neuroscience, Rehabilitation, Ophthalmology, Genetics, Maternal and Child Health, University of Genoa, 16132 Genoa, Italy; luca1puce@gmail.com (L.P.); ctrompetto@neurologia.unige.it (C.T.); 2 Jozef Pilsudski University of Physical Education in Warsaw, 00-968 Warsaw, Poland; piotr.zmijewski@insp.waw.pl; 3 Department of Food and Drugs, University of Parma, 43125 Parma, Italy; 4 IRCCS Ospedale Policlinico San Martino, 16132 Genoa, Italy

**Keywords:** pacing strategy, swimming, Down syndrome, autism spectrum disorder, middle-distance swimming, long-distance swimming, Virtus Global Games, World Aquatics Championships

## Abstract

Pacing strategy is a complex self-regulation process, crucial for optimising sports performance. Athletes with Intellectual Impairments (IIs) face unique challenges due to cognitive limitations that may hinder their ability to pace effectively, impacting chronometric performance. This study analysed the pacing profiles and chronometric performance across 253 event entries by elite swimmers with II, divided into three groups: 100 entries for group II1 (intellectual disability), 85 for group II2 (Down syndrome), and 68 for group II3 (autism spectrum disorder). These results were compared with 112 event entries from athletes without disabilities (AWDs). Data were collected from the 2023 Virtus Global Games and the 2023 World Aquatics Championships, focusing on middle-distance and long-distance events. Performance metrics were assessed using 50 m split times, and within-group variability was evaluated through coefficients of variation. Swimmers with IIs showed slower overall chronometric performance than AWDs, with the largest deficits observed in II2 athletes. The II1 and II3 groups displayed more comparable results, with the II1 group outperforming the others slightly. Despite the slower times, pacing profiles were largely similar across all groups, following a parabolic pacing strategy, especially for longer distances. Greater within-group variability in both chronometric performance and pacing profiles was observed in II2 and II3 athletes, reflecting higher functional heterogeneity. In contrast, II1 athletes, and even more so AWDs, exhibited more consistent performance and pacing across all events. While swimmers with II recorded slower times, their pacing strategies resembled those of AWDs, suggesting that cognitive limitations may not significantly impair pacing regulation in swimming. However, the higher variability in II2 and II3 athletes highlights the potential need for revised classification systems to ensure fair competition.

## 1. Introduction

The optimisation of sports performance in middle- and long-distance events requires the implementation of a meticulously devised pacing strategy that strikes a balance between speed, power, and energy distribution throughout the race [1,2,3]. An effective pacing strategy enables athletes to maximise energy output, ensuring that resources are optimally depleted by the race’s conclusion, while mitigating the risk of premature exhaustion [4]. Conversely, an improper pacing strategy results in an imbalanced energy utilisation, which limits endurance and, consequently, reduces the overall performance potential [5,6].

This process of self-regulation is influenced by a range of internal and external factors. Internally, pacing is governed by physiological, psychological, and cognitive mechanisms [7,8,9], while external conditions such as environmental variables (e.g., competitor presence) also play a significant role [10,11]. Cognitive skills, including the ability to interpret past experiences [3], assess personal capabilities, and respond to real-time race conditions, are critical to effective pacing [12]. Athletes must be able to accurately interpret fatigue signals, such as perceived effort [13] and its effects on performance [14]. Moreover, they must adapt their strategies in response to changing conditions and effectively respond to the tactics employed by competitors [11,15,16].

Research suggests that athletes with Intellectual Impairment (II), a broad classification that encompasses a variety of cognitive and developmental conditions, face unique pacing challenges [17]. Specifically, athletes in group II1—those with an intelligence quotient (IQ) ≤75 and associated deficits in adaptive behaviour [18]—have demonstrated difficulty maintaining a steady pace in activities such as submaximal running tests [19] and cycle ergometer tests [20]. In addition, during athletic events, these athletes often exhibit irregular pacing patterns compared to Athletes Without Disabilities (AWDs), who typically use more controlled and deliberate pacing strategies [21].

These pacing inconsistencies among athletes with II are largely attributed to cognitive limitations. In sports such as swimming, basketball, and table tennis, these athletes display significant deficits in sport-specific cognitive skills, including visual processing, reaction time, decision-making, short-term memory, and fluid reasoning [22]. These deficits, coupled with difficulties in accurately responding to environmental stimuli, can hinder performance and interaction with coaches, teammates, and opponents [23].

Moreover, certain forms of II, particularly those caused by genetic abnormalities and syndromes such as trisomy 21 (group II2 athletes) [24], are associated with physical disabilities. These include impaired motor coordination, reduced muscle control, lower cardiovascular endurance, and diminished strength [25,26], further widening the performance and pacing gap between athletes with II and their able-bodied peers.

Finally, athletes in the third and final group (II3), characterised as high-functioning individuals on the autism spectrum [24], also face challenges such as limited motor coordination and deficiencies in both fine and gross motor skills [27]. Additional difficulties with balance and motion planning are commonly reported in this group [28,29], further complicating their ability to sustain consistent pacing and optimal performance [30].

Despite the critical role of pacing in performance, research on pacing profiles in swimmers with II remains scarce, even though these athletes have shown significant technical and chronometric performance improvements in recent years [31]. Swimming, as a sport, presents a distinctive set of demands, requiring high levels of cognitive engagement for technical precision and coordination [32]. Unlike land-based sports such as running or cycling, swimmers contend with water resistance that is 600- to 1000-times greater than air resistance, resulting in significantly higher energy expenditure [33,34]. Mechanical efficiency in swimming is also relatively low, with only 6–18% of energy converted into propulsion, compared to 18–24% in cycling [35]. As a result, even small increases in swimming speed lead to disproportionately large increases in metabolic cost, making pacing strategies particularly crucial in middle- (200 m and 400 m) and long-distance events (800 m and 1500 m) [36]. In contrast, sprint events (50–100 m) follow an “all-out” pacing profile, where swimmers aim to reach peak velocity quickly and manage the inevitable decline due to fatigue [2].

Moreover, the aquatic environment isolates swimmers from many external stimuli—such as auditory and visual cues—that typically inform tactical decisions in other sports [24,37,38]. This isolation, combined with the complex interaction between physiological and cognitive demands, makes pacing in swimming particularly challenging for athletes with II.

The present study aims to analyse the pacing profiles, chronometric performance, and their within-group variability in elite swimmers with II in all three eligibility groups (II1, II2, and II3) compared to AWDs in middle- and long-distance events. The data, sourced from top-tier competitions representing the pinnacle of performance in their respective categories, were gathered from events conducted under uniform technical regulations to ensure a fair comparison.

Based on evidence from other sports such as cycling and athletics, and taking into account cognitive and physical limitations, we hypothesised that swimmers with II would exhibit more irregular profile patterns and lower overall chronometric performance, with more pronounced differences in long-distance events. In addition, given the heterogeneity of II in terms of cognitive and physical impairments, we expected greater within-group variability in outcome measures between groups.

## 2. Materials and Methods

### 2.1. Participants

This study focused on two distinct groups of athletes: a cohort of swimmers with II and a control group of AWDs, all of whom were finalists in international competitions during the summer of 2023. The II group was further divided into three eligibility subgroups: group II1, comprising individuals with an IQ of 75 or lower, significant limitations in adaptive behaviour (covering conceptual, social, and practical adaptive skills), and an impairment diagnosed before the age of 22, during their developmental years; group II2, consisting of individuals with Down syndrome (trisomy 21); and group II3, representing high-functioning individuals on the autism spectrum with an IQ above 75. In total, 365 event entries were analysed, spanning athletes from 81 countries. Within the II group, 100 entries were recorded for group II1 (21 countries), 85 entries for group II2 (18 countries), and 68 entries for group II3 (13 countries). The control group of AWDs accounted for 112 entries from 29 countries. All groups competed in the same seven events that were analysed, but differences in the number of entries arose because, in some finals, the number of participants from the II group was fewer than the maximum allowable eight athletes per event.

### 2.2. Procedure

This comparative experimental study sought to analyse pacing profiles, chronometric performance, and their within-group variability among athletes with II and their able-bodied counterparts. The experimental group consisted of athletes with II, divided into the three aforementioned eligibility groups, whose performance outcomes were recorded during the Virtus Global Games held in France from 5 to 9 June 2023. This event is recognised as one of the highest-level competitions for athletes with II, analogous to a world championship in this category, and adheres to rigorous competitive standards. Data from this event were publicly available and retrieved from the official website [39]. For the control group, the performances of able-bodied swimmers were analysed from the World Aquatics Championships, which took place in Fukuoka from 23 to 30 July 2023. Official data were obtained from the publicly accessible competition database [40].

The performance outcomes of both groups were analysed in the finals of middle- and long-distance swimming events that involved more than one participant. Specifically, this study included the 200 m butterfly, backstroke, breaststroke, and freestyle events, as well as the 400 m freestyle, for both male and female athletes. Long-distance events, such as the 800- and 1500 m freestyle, were also examined.

### 2.3. Ethical Considerations

As the data used in this study were publicly accessible and no direct interaction with human participants was required, ethical approval was not necessary [41]. However, all data were anonymised in compliance with established guidelines for the ethical use of publicly available datasets in research.

### 2.4. Sample Size a Priori Power Analysis

An a priori power analysis was not deemed necessary in this study due to the nature of the data collection process. Specifically, all data used in this analysis were systematically collected, meaning that data were gathered from a comprehensive and predefined source without selective sampling or exclusion of relevant cases. This systematic collection encompassed the entire population, which mitigates concerns about sample size sufficiency [42].

Chronometric performance was assessed by calculating the total race times, while pacing profiles were derived from the normalised 50 m split times, ensuring comparability across athletes, regardless of absolute speed differences.

Intra-group coefficients of variation were also calculated for both chronometric performance and pacing profiles to evaluate consistency and variability within each group.

### 2.5. Statistical Analysis

Descriptive statistics for pacing profiles and chronometric performance were reported as means and standard deviations, both overall and stratified by eligibility groups. Homogeneity of variances was tested using Levene’s test, while the normality of data distribution was assessed through the Shapiro–Wilk test. A one-way analysis of variance (ANOVA) was conducted for pacing profiles at each split time, followed by the Games–Howell post hoc test to adjust for unequal variances and unequal sample sizes. The same analytical approach was applied to chronometric performance. A significance threshold of *p* < 0.05 was adopted, with adjustments for multiple comparisons as needed. All statistical analyses were performed using the open-source Jamovi software (Version 2.5) (The Jamovi project, 2024), available at https://www.jamovi.org, accessed on 22 October 2024.

## 3. Results

### 3.1. Chronometric Performance

**Figure 1:**
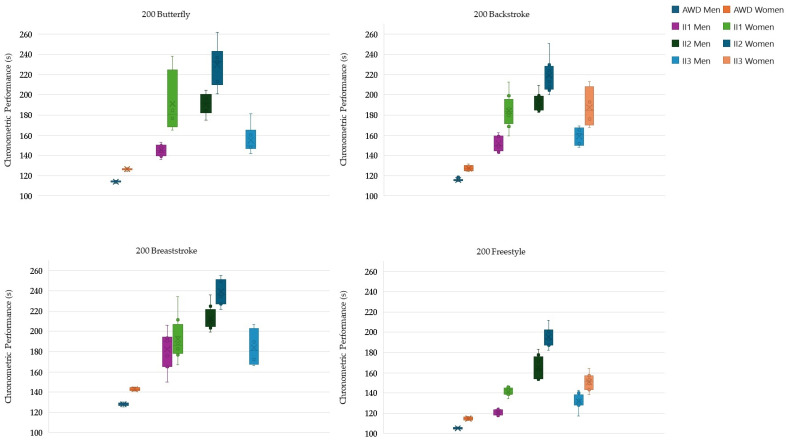
Box plots showing chronometric performance (total race time) for the 200 m events (butterfly, backstroke, breaststroke, and freestyle) for men and women athletes from all four groups (AWDs, II1, II2, and II3). AWD: athlete without disability, II1: athletes with intellectual disability (IQ ≤ 75), II2: athletes with Down syndrome, II3: athletes with autism spectrum disorder.

**Figure 2:**
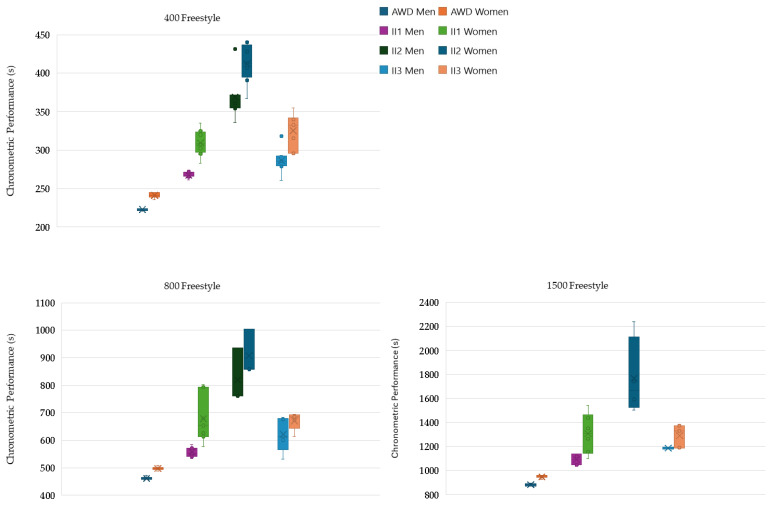
Box plots showing chronometric performance (total race time) for the 400 m, 800 m, and 1500 m freestyle events for men and women athletes from all four groups (AWDs, II1, II2, and II3). AWD: athlete without disability, II1: athletes with intellectual disability (IQ ≤ 75), II2: athletes with Down syndrome, II3: athletes with autism spectrum disorder.

Analysis of chronometric performance between the different groups and swimming events revealed several significant patterns, particularly when comparing the AWDs with the other groups (Figure 1 and Figure 2).

In the men’s 200 m butterfly, AWDs performed better than II1 (mean difference, MD = −27.52%, *p* < 0.001), II2 (MD = −67.35%, *p* < 0.001), and II3 (MD = −36.04%, *p* = 0.003). II1 performed better than II2 (MD = −31.24%, *p* < 0.001), whereas II3 performed worse than II2 (MD = 18.71%, *p* = 0.003). For women, AWDs performed better than II1 (MD = −51.14%, *p* = 0.055) and II2 (MD = −81.67%, *p* < 0.001).

In the men’s 200 m backstroke, AWDs performed better than II1 (MD = −31.01%, *p* < 0.001), II2 (MD = −66.35%, *p* < 0.001), and II3 (MD = −37.23%, *p* = 0.001). II1 performed better than II2 (MD = −26.91%, *p* < 0.001), whereas II2 performed worse than II3 (MD = 37.23%, *p* < 0.001). In women, AWDs performed better than II1 (MD = −44.47%, *p* < 0.001), II2 (MD = −72.15%, *p* < 0.001), and II3 (MD = −46.8%, *p* = 0.023), while II1 performed better than II2 (MD = −19.16%, *p* = 0.004).

In the men’s 200 m breaststroke, AWDs achieved better times than II1 (MD = −42.24%, *p* = 0.002), II2 (MD = −66.11%, *p* < 0.001), and II3 (MD = −43.46%, *p* = 0.035). II1 performed better than II2 (MD = −16.78%, *p* = 0.031). In women, AWDs performed better than II1 (MD = −34.66%, *p* < 0.001) and better than II2 (MD = −67.70%, *p* < 0.001), while II1 performed better than II2 (MD = −24.54%, *p* < 0.001).

In the men’s 200 m freestyle, significant differences were observed on all fronts. AWDs performed better than II1 (MD = −14.91%, *p* < 0.001), II2 (MD = −56.89%, *p* < 0.001), and II3 (MD = −24.93%, *p* < 0.001). II1 had better times than II2 (MD = −36.53%, *p* < 0.001) and II3 (MD = −8.72%, *p* = 0.025), while II3 performed better than II2 (MD = −30.36%, *p* < 0.001). In the women, AWDs performed better than II1 (MD = −22.88%, *p* < 0.001), II2 (MD = −70.18%, *p* < 0.001), and II3 (MD = −31.52%, *p* < 0.001), while II1 performed better than II2 (MD = −38.50%, *p* < 0.001), and II3 performed better than II2 (MD = −51.19%, *p* < 0.001).

In the men’s 400 m freestyle, AWDs performed better than II1 (MD = −19.99%, *p* < 0.001), II2 (MD = −65.53%, *p* < 0.001), and II3 (MD = −28.52%, *p* < 0.001). II1 had better times than II2 (MD = −37.95%, *p* < 0.001) and II3 (MD = −7.11%, *p* = 0.049), while II2 performed worse than II3 (MD = 22.37%, *p* < 0.001). For women, AWDs performed better than II1 (MD = −28.25%, *p* < 0.001), II2 (MD = −70.89%, *p* < 0.001), and II3 (MD = −34.75%, *p* < 0.001). II1 performed better than II2 (MD = −33.24%, *p* < 0.001), while II2 performed worse than II3 (MD = 21.15%, *p* < 0.001).

In the men’s 800 m freestyle, AWDs achieved better times than II1 (MD = −20.60%, *p* < 0.001) and II3 (MD = −34.55%, *p* = 0.015). For women, AWDs performed better than II1 (MD = −36.46%, *p* = 0.006), II2 (MD = −82.44%, *p* = 0.034), and II3 (MD = −35.01%, *p* < 0.001). A marginally significant result was observed between II1 and II2 (MD = −33.69%, *p* = 0.058).

In the 1500 m freestyle for men, AWDs performed better than II1 (MD = −23.64%, *p* < 0.001) and II3 (MD = −34.44%, *p* < 0.001), while II1 performed better than II3 (MD = −8.73%, *p* = 0.004). For women, AWDs achieved better results than II2 (MD = −86.32%, *p* = 0.045) and II3 (MD = −36.07%, *p* = 0.004) and better times than II1 (MD = −38.03%, *p* = 0.012).

### 3.2. Coefficient of Variation

The comparison of CV in timing and pacing between the groups, as reported in Table 1, revealed distinct patterns of variability among the groups for different events.

### 3.3. Pacing Profile

**Figure 3:**
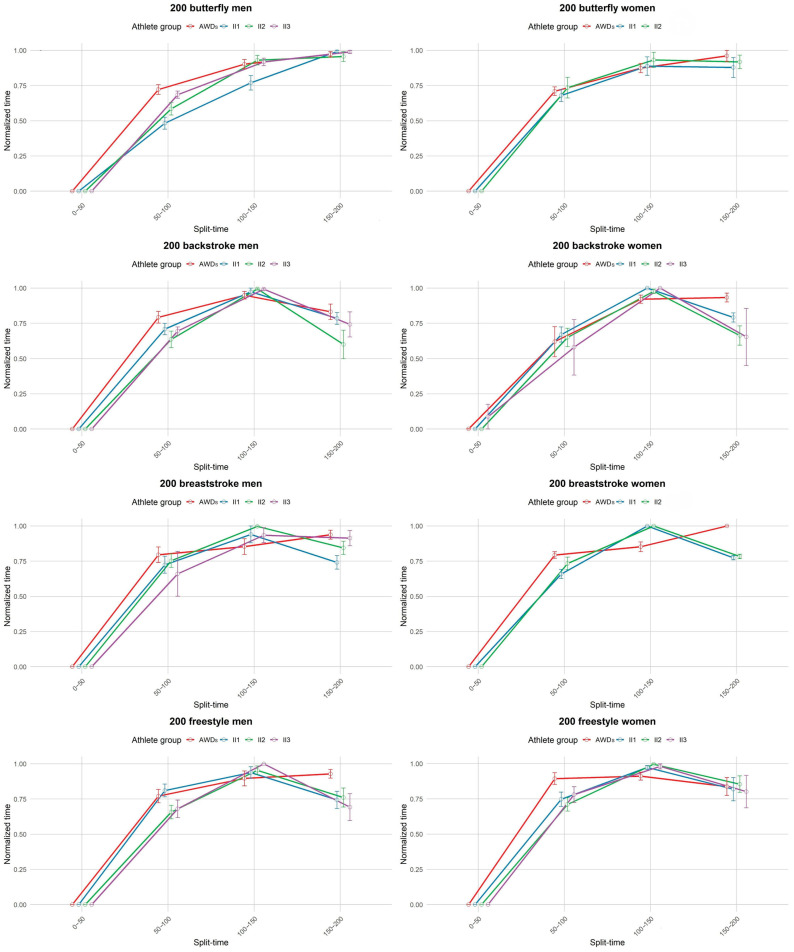
Pacing profile showing the split time (0–50 m) for the 200 m events (butterfly, backstroke, breaststroke, and freestyle) for men and women athletes from all four groups (AWDs, II1, II2, and II3). AWDs: athletes without disability, II1: athletes with intellectual disability (IQ ≤ 75), II2: athletes with Down syndrome, II3: athletes with autism spectrum disorder.

**Figure 4:**
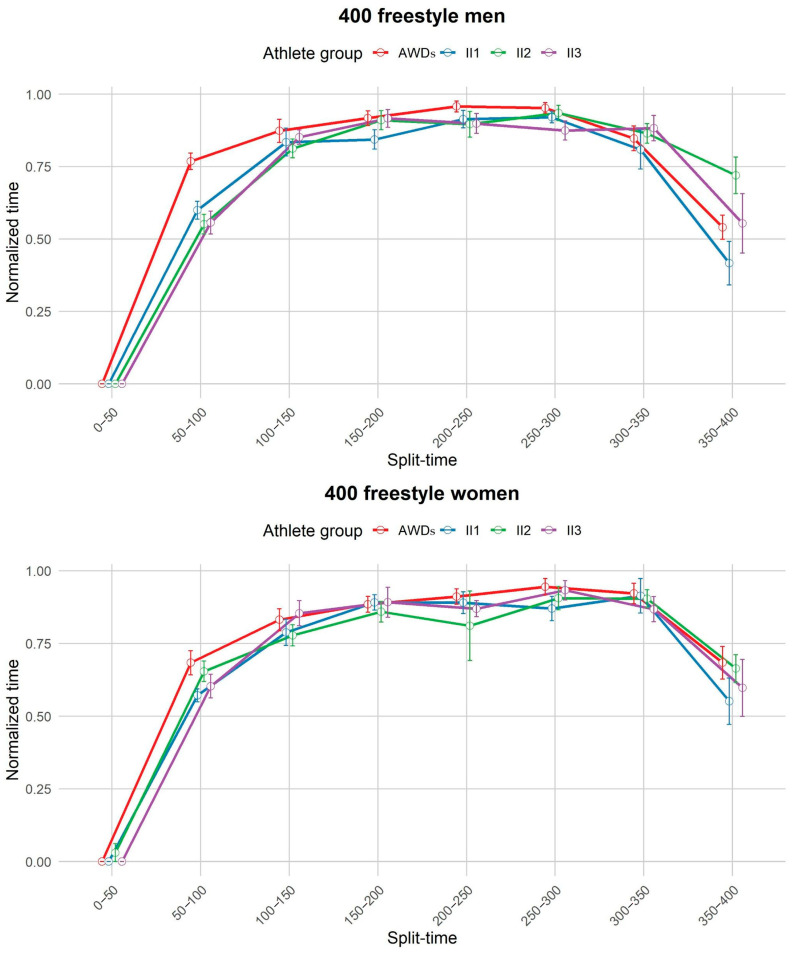
Pacing profile showing the split time (0–50 m) for the 400 m freestyle events for men and women athletes from all four groups (AWDs, II1, II2, II3). AWDs: athletes without disability, II1: athletes with intellectual disability (IQ ≤ 75), II2: athletes with Down syndrome, II3: athletes with autism spectrum disorder.

**Figure 5:**
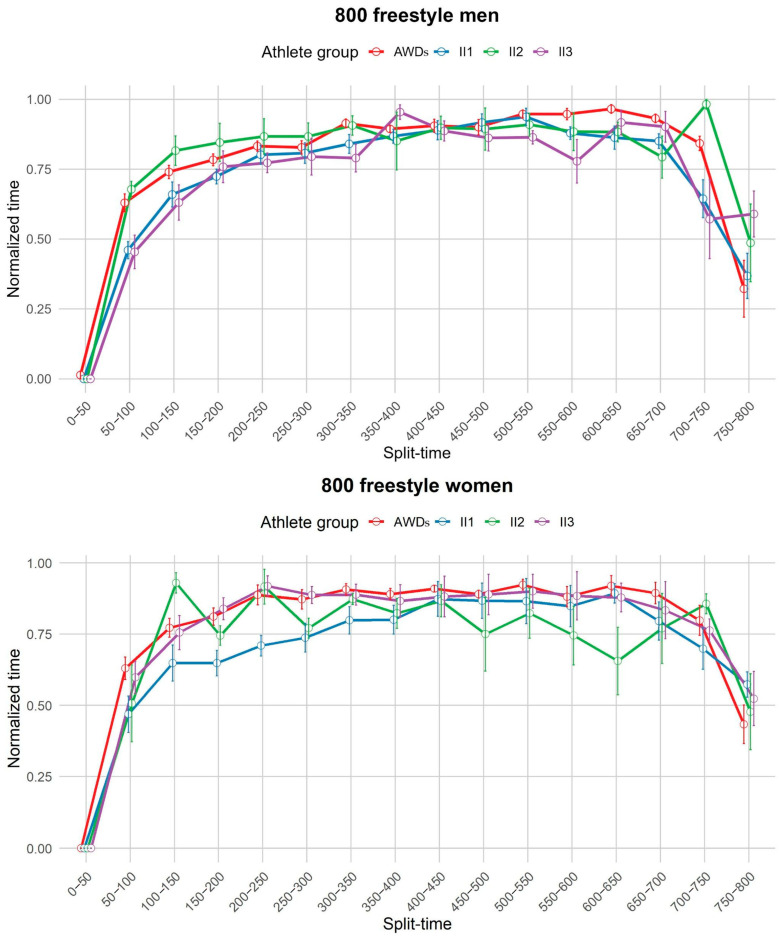
Pacing profile showing the split time (0–50 m) for the 800 m freestyle events for men and women athletes from all four groups (AWDs, II1, II2, II3). AWDs: athletes without disability, II1: athletes with intellectual disability (IQ ≤ 75), II2: athletes with Down syndrome, II3: athletes with autism spectrum disorder.

**Figure 6:**
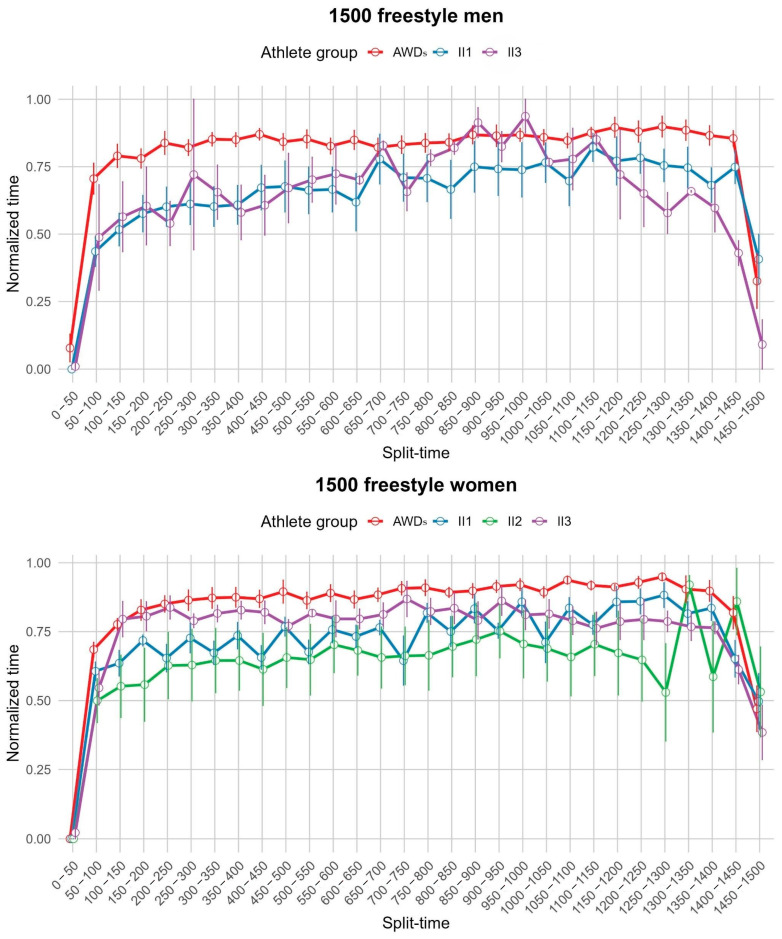
Pacing profile showing the split time (0–50 m) for the 1500 m freestyle events for men and women athletes from all four groups (AWDs, II1, II2, II3). AWDs: athletes without disability, II1: athletes with intellectual disability (IQ ≤ 75), II2: athletes with Down syndrome, II3: athletes with autism spectrum disorder.

Qualitative analysis of the pacing profiles between groups revealed a similar parabolic pattern in all groups: a fast start, a steady or slightly decreasing pace in the middle, followed by a strong finish. This pattern was particularly pronounced in long-distance races, whereas in shorter races (200 m) the final sprint showed more variability both between events and between groups. At these shorter distances, group II performed better in the final sprint than the AWDs group (Figure 3, Figure 4, Figure 5 and Figure 6).

Objective analysis of pacing profiles between the groups revealed significant differences in only a few segments of certain swimming events.

In the women’s 200 m breaststroke, AWDs swam at a slower pace than II1 during the 50–100 m segment (MD = 0.135, *p* = 0.014). However, AWDs swam faster than both II1 and II2 in the 100–150 m segment (MD = −0.148, *p* = 0.009 for both comparisons). During the final 150–200 m segment, AWDs were slower than both II1 (MD = 0.226, *p* < 0.001) and II2 (MD = 0.21752, *p* < 0.001).

In the men’s 400 m freestyle, AWDs paced slower than II1 (MD = 0.169, *p* = 0.006), II2 (MD = 0.2173, *p* = 0.001), and II3 (MD = 0.21191, *p* = 0.004) in the early stages (50–100 m). In the final stages (350–400 m), II1 accelerated compared to II2 (MD = −0.304, *p* = 0.036), indicating a less rapid arrival for II2.

In the men’s 800 m freestyle, both AWDs and II2 swam slower than II1 in the early stages (50–100 m) (AWDs vs. II1: MD = 0.16965, *p* = 0.009; II2 vs. II1: MD = 0.21789, *p* = 0.006). In the later stages (700–750 m), II2 slowed down compared to both AWDs (MD = 0.14022, *p* = 0.007) and II1 (MD = 0.33824, *p* = 0.006), who increased their pace. In the women’s 800 m freestyle, II2 swam at a slower pace than II1 between 100–150 m (MD = 0.282, *p* = 0.020). During the 150–200 m segment, AWDs were slower than II1 (MD = 0.163, *p* = 0.048), and II3 also paced slower than II1 (MD = 0.378, *p* = 0.038). This trend continued in the 200–250 m segment, where AWDs again swam slower than II1 (MD = 0.178, *p* = 0.017), with II3 trailing as well (MD = 0.149, *p* = 0.010).

In the men’s 1500 m freestyle, AWDs paced slower than II3 during the mid-early stages (600–650 m) (MD = 0.149, *p* = 0.012). In the final stages, significant differences emerged, with II3 swimming faster in the 1000–1050 m segment (MD = −0.092, *p* = 0.042), slower in the 1300–1350 m segment (MD = 0.2266, *p* = 0.001), and faster again in the 1400–1450 m segment (MD = −0.425, *p* < 0.001). II1 also paced slower than II3 in the final 1400–1450 m segment (MD = 0.319, *p* = 0.017). In the women’s 1500 m freestyle, AWDs paced slower than II1 in several segments of the first half (200–700 m), including 200–250 m (MD = 0.197, *p* = 0.037), 300–350 m (MD = 0.199, *p* = 0.020), and 500–550 m (MD = 0.187, *p* = 0.023). This slower pace from AWDs persisted in the 650–700 m segment (MD = 0.119, *p* = 0.028). In the later stages (900–1150 m), AWDs continued to swim slower than II1, particularly in the 900–950 m segment (MD = 0.161, *p* = 0.004) and the 1100–1150 m segment (MD = 0.143, *p* = 0.026). Furthermore, AWDs were slower than II3 in the 1250–1300 m segment (MD = 0.162, *p* = 0.033).

### 3.4. Post Hoc Power Analysis

A post hoc power analysis was conducted to evaluate the adequacy of the sample sizes used in this study for detecting the observed differences. The analysis utilised the sample sizes for each group (AWDs: 112, II1: 100, II2: 85, II3: 68) and the observed effect sizes (e.g., AWDs vs. II1: 27.52%; AWDs vs. II2: 67.35%). At a significance level of α = 0.05, the results showed that the overall power was adequate, exceeding 80% for comparisons with larger observed effect sizes, confirming the sufficiency of the sample sizes to detect significant differences.

## 4. Discussion

By comparing pacing profiles, chronometric performance, and their variability among the three ability groups and AWDs, this study aimed to elucidate the impact of II on middle- and long-distance swimming events. As expected, athletes with II underperformed compared to their non-disabled peers, with II2 athletes exhibiting the lowest chronometric performances. Athletes in the II1 and II3 groups showed more comparable results, with II1 generally performing slightly better. For instance, in 200 m events, performance differences between AWDs and II2 ranged from 57% to 82%, with the smallest gap in the men’s 200 m freestyle and the largest in the women’s 200 m butterfly. Similarly, differences between AWDs and II1 spanned from 15% to 51%, while for AWDs and II3, the gap ranged from 25% to 47%.

Comparable trends were seen in the 400 m freestyle, with AWDs outperforming II2 by 66% for men and 71% for women. AWDs-II1 differences were smaller, ranging from 20% to 28%, while AWDs-II3 differences hovered around 29% to 35%. These differences did not expand in longer distances (800 m and 1500 m), indicating that performance gaps remained relatively consistent across distances. For example, the AWDs-II2 gap remained between 77% and 86%, while AWDs-II1 showed a range of 21% to 38%. The AWDs-II3 gap remained constant at approximately 35% to 36%.

These findings are consistent with previous studies in other sports that have highlighted the challenges that athletes with II face in maintaining competitive performance. For example, Andrews, Goosey-Tolfrey, and Bressan [43] found a significant reduction in speed and acceleration in runners with II compared to their nondisabled counterparts. In addition, comparisons of world athletics records showed that the smallest differences in performance were observed in sprint and middle-distance events, with differences of about 10% for men and 15% for women. However, in long-distance events, the gap widened to 18% for men and 23% for women. In events such as jumping, the differences increased even more, with a gap of up to 20% for men and 24% for women. Throwing events showed the largest performance differences, with gaps exceeding 40% for both genders, except for shooting [44]. These findings reinforced the idea that II was a significant barrier to athletic performance in various disciplines, although the impact varied depending on the type of event, distance, and, in the case of our study, the specific type of II.

While performance differences between able-bodied athletes and those with II were evident, our study found minimal discrepancies in pacing profile between the groups. Contrary to expectations, pacing profiles for swimmers with II were largely consistent with those of their able-bodied counterparts, particularly in longer-distance events. These differences were mainly confined to specific split times of certain events and did not significantly impact overall pacing patterns. Qualitative analyses also revealed that the overall pacing profiles between the two groups remained surprisingly similar. In the 200 m, for example, pacing strategies commonly followed a parabolic pattern: a fast start, a steady or slightly decreasing pace in the middle, and a strong finish. However, the strong finish was only present in certain events and was more pronounced in group II. This parabolic trend was even more pronounced in longer distances like the 1500 m, where some swimmers—both able-bodied and those with II—demonstrated an ability to swim the second half of the race either at the same pace or faster than the first half [36,45].

This observation contrasts with previous research on land-based sports, where athletes with II often exhibit irregular pace profiles. For instance, in running, individuals with II commonly struggle to maintain consistent submaximal speeds over 400 m, frequently accelerating towards the end, in contrast to the steadier pacing observed in nondisabled runners [19]. Similarly, in cycling, maintaining a predetermined submaximal speed is a noted challenge for athletes with II [20]. Evidence further indicates a pattern of slower starts, mid-race acceleration, and deceleration near the finish line in runners with II during both 400 m and 1500 m events [21]. These findings suggest that the cognitive demands associated with pacing and energy regulation play a significant role in the observed performance profiles of athletes with II in land sports.

However, swimming may provide a unique context that mitigates these challenges. The repetitive and cyclical nature of swimming movements, combined with the sensory isolation offered by the aquatic environment, appears to significantly reduce the cognitive load required to maintain a consistent pace [37,38]. This could explain the unexpected findings of this study: unlike in land-based sports, swimmers with II demonstrated greater pacing regularity across events, and, despite performing below their able-bodied counterparts, the performance gaps remained surprisingly consistent even in longer distances. Furthermore, evidence suggests that with appropriate instruction and tailored training programs—characterised by high repetition, targeted feedback, and sufficient practice time, which are typical components of training for swimmers with disabilities [5]—athletes with II can reach performance levels similar to those of their typically developing peers [46,47,48,49].

Given the similarity in pacing profiles, it is clear that the significant differences in chronometric performance between athletes with II and AWDs are due to other factors independent of cognitive limitations. One critical element is access to advanced training methodologies. While non-disabled athletes typically benefit from highly specialised coaching and comprehensive sports science support—including nutritionists, sports psychologists, and strength coaches—athletes with II often lack access to equivalent resources, which limits their ability to optimise key performance factors such as speed, endurance, and recovery [50,51,52]. Additionally, disparities in training infrastructure play a significant role; elite athletes typically train in state-of-the-art facilities, whereas athletes with II may have to contend with suboptimal conditions, further hindering their performance potential [53]. This underlines the absolute importance of providing comprehensive support in preparing athletes for competition and ensuring that they receive adequate support that goes beyond simply measuring their performance. Addressing these inequalities is essential to fostering an equitable environment in which all athletes could reach their full potential.

The final aim of this study was to examine between-group variability in timing and pacing, key elements in assessing competitive equity among athletes with disabilities. Higher within-group variability may indicate that although athletes meet medical eligibility criteria, there are significant differences in the level of functionality required by the sport. Such inhomogeneities could affect the fairness of competitions [54]. The results indicate that group II1 has moderate variability in timing and pacing, which ensures balanced competition. This group occupies an intermediate position between the AWDs and II2–3 groups. On the other hand, groups II2 and II3 show greater variability, particularly in long-distance events and more complex strokes, suggesting the need to revise their classification to improve fairness. Competition organisers for this purpose could consider incorporating additional classification criteria that take into account functional variability within these groups. These adjustments would not only optimise athletic development, but also better align competitive standards with the different needs and abilities of these athletes. Specifically, the high variability in group II2 may be due to a combination of cognitive and physical challenges typical of Down syndrome, such as reduced cardiovascular endurance, decreased muscle strength, and motor coordination problems, which manifest themselves with varying intensity among athletes [25,26]. Group II3, composed of athletes with autism spectrum disorders, has significant functional heterogeneity as symptoms and skill levels vary widely, further complicating the maintenance of fairness in competitions [55,56].

This study is not without limitations. The relatively small sample size, especially in the II2 group, restricts the generalisability of our findings. While this study provides valuable insights into pacing profiles in sprint events, the extent to which these findings can be extrapolated to other sports or levels of competition remains uncertain. Differences in physiological demands, tactical elements, and environmental factors in other sports or at different levels of performance may limit the applicability of these results. Additionally, split times were measured in 50 m intervals, which, while suitable for analysing overall pacing patterns, may lack the resolution needed for shorter events like the 200 m. More granular data—such as split times at 10 m or 25 m intervals—could provide deeper insights into the finer dynamics of pacing strategies, particularly in sports or events with rapid changes in velocity or effort.

## 5. Conclusions

This study compared pacing profiles, chronometric performance, and within-group variability among elite swimmers with II and able-bodied athletes (AWDs) in middle- and long-distance events. Swimmers with II exhibited slower chronometric performance compared to AWDs, with the largest deficits observed in athletes with Down syndrome (II2), while groups II1 and II3 demonstrated more comparable results. Despite these differences, swimmers with II maintained regular pacing patterns similar to AWDs, even in long-distance events. This contrasts with land-based sports, where athletes with II often show less regular pacing profiles than their able-bodied counterparts, alongside performance gaps that typically widen with increasing distance.

Regarding within-group variability, groups II2 and II3 showed the highest levels, particularly in long-distance events, reflecting substantial differences in performance among athletes within these groups. In contrast, group II1 displayed more consistent results, with performance closer to that of AWDs in terms of pacing stability. These findings underscore the functional diversity present within groups II2 and II3.

The findings highlight the potential of swimming as a supportive environment that mitigates some cognitive and physical challenges observed in land-based sports, while also revealing areas where classification systems may need refinement to account for the broad functional diversity within groups.

Future research should seek to include larger, more diverse cohorts of athletes with II sampled from more international events to refine our understanding of pacing strategies and performance variability across impairment groups. Additionally, longitudinal studies would allow for tracking the evolution of pacing strategies as athletes gain experience and improve with targeted training.

## Figures and Tables

**Table 1:** Coefficients of variation (CVs) for timing and pacing across events in men (M) and women (W). AWDs: athletes without disability, II1: athletes with intellectual disability (IQ ≤ 75), II2: athletes with Down syndrome, II3: athletes with autism spectrum disorder.

EVENT	SEX	CV TIMING				CV PACING			
		AWDs	II1	II2	II3	AWDs	II1	II2	II3
200 Butterfly	M	0.8	4.2	5.8	9.1	1.4	4.7	6.1	9.2
	W	1	16.8	9.1	-	1.7	16.9	9.8	-
200 Backstroke	M	1.2	4.9	4.8	5.6	1.6	5.4	5.2	5.8
	W	2.1	9.0	7.5	10.6	2.6	9.1	7.8	10.9
200 Breaststroke	M	1.2	10.7	5.6	10.0	2.0	11.1	5.8	9.8
	W	1.2	11.1	5.4	-	1.4	11.2	5.6	-
200 Freestyle	M	0.8	2.1	6.9	5.9	1.4	2.6	7.8	6.3
	W	1.4	2.9	5.2	5.7	1.7	3.3	5.8	6.2
400 Freestyle	M	0.7	1.5	7.5	5.6	1.2	2.4	8.1	6.0
	W	1.5	5.4	6.1	7.2	1.8	5.7	6.7	7.5
800 Freestyle	M	1.3	3.2	12.3	9.9	1.5	3.7	12.4	10.1
	W	1.2	13.0	9.3	5.0	11.6	19.3	18.3	15.8
800 Freestyle	M	1.3	3.2	12.3	9.9	1.5	3.7	12.4	10.1
	W	1.2	13.0	9.3	5.0	11.6	19.3	18.3	15.8
1500 Freestyle	M	2.2	8.1	-	3.7	1.4	7.3	-	3.7
	W	1.3	12.9	18.5	7.4	1.5	13.0	18.6	7.7

## Data Availability

Publicly available datasets were analyzed in this study. This data can be found here: https://gg2023.org/en/sports/swimming-en/ (accessed on 22 October 2024), https://www.worldaquatics.com/competitions/1/world-aquatics-championships-fukuoka-2023/results?disciplines=SW (accessed on 22 October 2024).
